# Introduction to Editorial Board Member: Professor Jennifer L. West

**DOI:** 10.1002/btm2.10225

**Published:** 2021-05-10

**Authors:** Emily S. Day

**Affiliations:** ^1^ Department of Biomedical Engineering University of Delaware Newark Delaware USA; ^2^ Department of Materials Science and Engineering University of Delaware Newark Delaware USA; ^3^ Center for Translational Cancer Research, Helen F. Graham Cancer Center and Research Institute Newark Delaware USA

We are pleased to introduce Editorial Board Member Professor Jennifer L. West in this issue of *Bioengineering and Translational Medicine* (Figure 1). Prof. West will be Dean of Engineering and Applied Science at the University of Virginia (UVA) effective July 1, 2021, becoming the first woman to lead UVA Engineering.^1^ Currently, she is the Fitzpatrick Family University Distinguished Professor of Engineering at Duke University, where she also serves as Associate Dean for Ph.D. Education. In addition to her primary faculty appointment in Biomedical Engineering, Prof. West holds appointments in Mechanical Engineering and Materials Science, Cell Biology, and Chemistry. She is also Core Faculty in Innovation and Entrepreneurship, a Member of the Duke Cancer Institute, and an Affiliate of the Regeneration Next Initiative. Before moving to Duke University in 2013, Prof. West was the Isabel C. Cameron Professor of Bioengineering at Rice University, where she began her academic career in 1996 after earning her doctorate from the University of Texas at Austin. She also served as Bioengineering Department Chair during her time at Rice. Over the years, West has garnered international acclaim for her contributions to the fields of biomaterials, tissue engineering, and nanomedicine.

**FIGURE 1:**
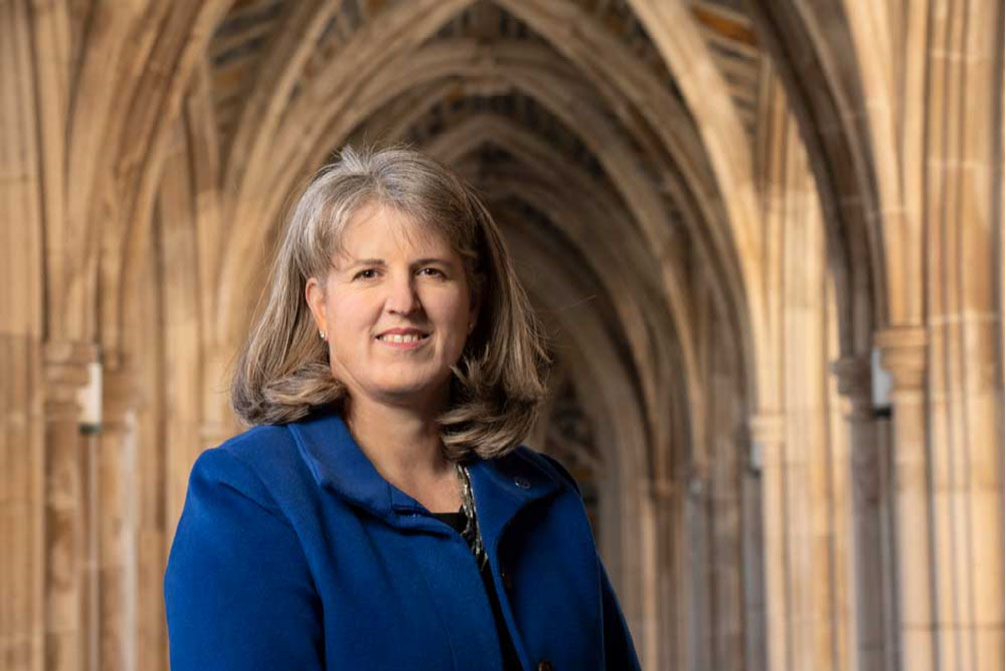
Professor Jennifer L. West at Duke University. Photo credit: Duke Graduate School

During her tenure at Rice University, West pioneered the development of nanoparticle‐mediated photothermal therapy together with Naomi Halas, another Rice professor. In 2003, they reported that nanoshells composed of 120 nm silica cores and 15‐nm thick gold shells could be delivered into tumors and irradiated with tissue‐penetrating near‐infrared light, causing the nanoshells to produce heat sufficient to induce thermal tumor damage.^2^ This seminal paper has been cited >4200 times, and led researchers worldwide to begin investigating photothermal therapy as a treatment for various cancer subtypes. West and Halas co‐founded Nanospectra Biosciences to facilitate the clinical translation of nanoshell‐mediated photothermal therapy. The results of a clinical trial in patients with low‐ or intermediate‐risk localized prostate cancer were published in late 2019 and validated the feasibility and safety of this treatment regimen.^3^ For their invention, West and Halas were named co‐recipients of the Best Discovery of 2003 Award from Nanotechnology Now and they also were named 2005 Outstanding Women of Achievement in Science and Technology by the YWCA of Houston.^4^, ^5^ Since the original breakthrough of photothermal therapy, West has further contributed to advancements in nanomedicine by developing numerous innovative nanotechnologies for multi‐modal imaging enhancement, light‐responsive drug delivery, and more.^6^, ^7^, ^8^, ^9^, ^10^, ^11^, ^12^, ^13^, ^14^, ^15^, ^16^

Beyond her contributions to nanomedicine, Prof. West is also known for her pioneering research in biomaterials engineering. In this regard, she creates unique biofunctional materials and incorporates these materials in tissue engineering applications. In particular, West is known for her work designing extracellular matrix (ECM)‐mimetic hydrogels, which support cell encapsulation to enable regenerative medicine applications.^17^, ^18^, ^19^ She has invented novel microfabrication strategies for three‐dimensional (3D) biomimetic patterning that enable researchers to recapitulate with high precision the *in vivo* vascular architecture of desired organs.^20^ Using specifically tailored hydrogels, her lab has also performed research that provides insight to the role of specific cells (e.g., adipose‐derived stem cells, macrophages), growth factors, or peptides on vascularization.^21^, ^22^, ^23^ Finally, West has also led the development of two‐dimensional biomimetic platforms that enable studies of cell biology and stem cell differentiation.^24^, ^25^, ^26^, ^27^ Her impact in the field of biomaterials and tissue engineering is substantial, as her work spanning these fields and nanotechnology has earned over 50,000 citations to date.

For her scientific achievements, Prof. West has received numerous awards and honors. She is a member of the National Academy of Engineering and the National Academy of Inventors, and a Fellow of the American Institute for Medical and Biological Engineering (AIMBE) and of the Biomedical Engineering Society (BMES). West is a Howard Hughes Medical Institute Professor and in 2015 she received the Clemson Award from the Society for Biomaterials, which is bestowed upon those who have advanced knowledge of material/tissue interactions. In 2017, she was an invited lecturer at the annual meeting of the President's Circle, an honorary association that engages with the Presidents of the National Academies of Science, Engineering, and Medicine to ensure they have the necessary resources to further their work.

In addition to her scientific accolades, Prof. West is also known for her outstanding teaching and mentoring. At Duke University, West has been honored with the 2019 Dean's Award for Excellence in Mentoring and with the 2016 Capers and Marion McDonald Award for Excellence in Teaching and Research.^28^, ^29^ While at Rice, Prof. West received the Excellence in Research Mentorship Award as well as the J.M. Chance Prize for Excellence in Teaching.^30^ Her trainees have advanced to prominent careers in academia and industry, and many attribute her mentorship to their success. In support of her nomination for the 2019 Dean's Award for Excellence in Mentoring, one of West's former trainees stated, “Dr. West possesses the power to encourage in exacting detail. It is almost as if she knows each person's weaknesses or fears and takes great care with specific effort to have them addressed…Dr. West took me under her wing and told me I could fly. When I didn't believe her, she pushed me out of the proverbial ‘nest’ and now, I'm flying.”^28^ Another wrote, “I will always look up to Dr. West as a scientist and mentor. Her knowledge, insight, and integrity are admirable. She cares about her students, both personally and professionally. She always believed in me and sought out opportunities for my growth.”^28^

These sentiments were echoed by other alumni of the West Lab who were contacted for comment. They described Prof. West as “an exemplary leader and mentor…who is always willing to share her experience and knowledge to guide her trainees” and as someone who “not only promotes the accomplishments of her own trainees but is more broadly dedicated to championing people and raising others up. She is an exemplar of generosity and positivity.” Many noted that Prof. West's guidance extends well beyond the years her students work in the lab, and that she is unwavering in her support. One alumna stated, “It was her confidence in me that enabled me to develop confidence in myself as a scientist.” On behalf of all of Prof. West's former and current mentees, I thank her for her moral support, guidance, and advocacy, which has positively impacted numerous individuals and contributed greatly to their success.

Finally, Prof. West has performed outstanding service to the profession. She has served on the editorial board of numerous scientific journals and participated in several grant review panels for the NIH and NSF. She was Treasurer of BMES from 2011 to 2015 and served on the BMES Diversity Committee from 2014 to 2017. Prof. West was Vice Chair and Chair Elect of the Gordon Research Conference on Biomaterials and Tissue Engineering from 2015 to 2019 (Figure 2) and Chair of the National Academy of Engineering Frontiers of Engineering Symposium from 2016 to 2019. Furthermore, she was Chair of the AIMBE College of Fellows from 2013 to 2015 and President Elect of the North Carolina Tissue Engineering and Regenerative Medicine Society from 2016 to 2018. Overall, West's dedicated leadership and impactful service have greatly advanced the bioengineering community over the years.

**FIGURE 2:**
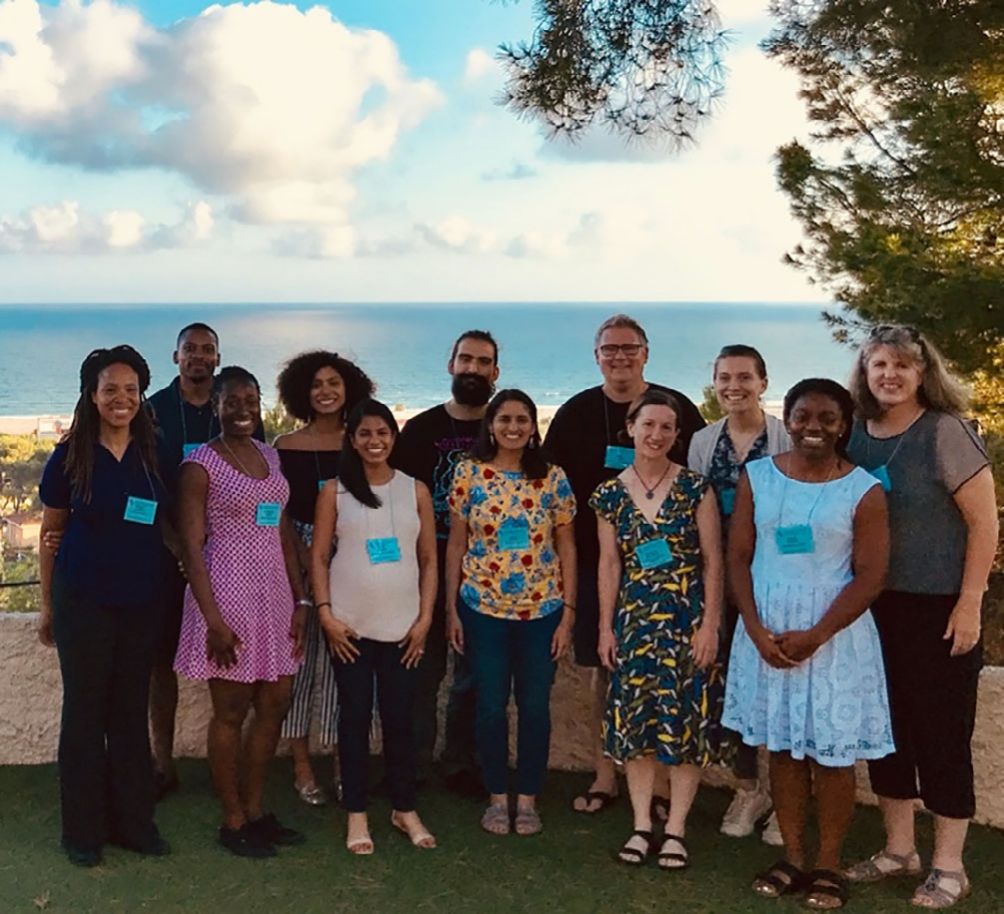
West Lab members and alumni at the 2019 Gordon Research Conference on Biomaterials and Tissue Engineering

Prof. West has had an immense impact on biomaterials and nanomedicine research, and on the field of biomedical engineering more broadly. She is a remarkable leader who has nurtured the careers of many in the field, including both former trainees and those not affiliated with her lab. On behalf of the scientific community, I am honored to thank her for her research contributions, selfless service, and dedication to inspiring and empowering others through meaningful mentorship.

## AUTHOR CONTRIBUTIONS

**Emily Day:** Conceptualization; writing‐original draft; writing‐review & editing.

### PEER REVIEW

The peer review history for this article is available at https://publons.com/publon/10.1002/btm2.10225.
